# Design of a Cylindrical Compliant Linear Guide with Decoupling Parallelogram Mechanisms

**DOI:** 10.3390/mi13081275

**Published:** 2022-08-08

**Authors:** Tinghao Liu, Guangbo Hao

**Affiliations:** Electrical and Electronic Engineering, School of Engineering and Architecture, University College Cork, T12 K8AF Cork, Ireland

**Keywords:** compliant mechanism, linear guide, decoupling mechanism, finite element analysis

## Abstract

A conventional linear guiding mechanism refers to the slide rail guides composed of multiple assemble parts. These guiding mechanisms suffer from many adverse effects, including lubrication, wear and assembly issues. A novel compliant guiding mechanism is proposed in this paper to address these common problems, and this mechanism transfers or transforms motion, force and energy via the deformation of flexible members. This linear guide is designed in a cylindrical shape, and the centre platform moves along its axis (i.e., the motion direction). The proposed linear guide consists of several in-parallel curved compound double parallelogram mechanisms (CDPMs) connected by the same number of decoupling parallelogram mechanisms. Nonlinear finite element analysis (FEA) is used for stiffness analysis and shows that applying the decoupling mechanisms to the detached linear guide (the in-parallel curved CDPMs only) can dramatically improve the stiffness in undesired movement (bearing) directions while keeping its original stiffness along its axis. The nonlinear FEA can capture the stiffness variation by considering all the structural deformation. The issue of bearing-direction stiffness degradation of the detached linear guide is dealt with by applying decoupling mechanisms. The static experimental test is conducted on a 3D printed prototype and shows that the stiffness in the motion direction is nearly constant (linear). The results obtained from the experimental test show good agreement with those obtained from the nonlinear FEA with a maximum error of 9.76%.

## 1. Introduction

Linear guiding mechanisms have attracted significant attention from both manufacturing industries and researchers [1]. Traditional linear positioning is achieved by using slide rail guides. These linear guides are generally composed of multiple assemble parts, including railways and rods [2]. Although the conventional linear guides benefit from their relatively extensive range of motion, they suffer from many adverse aspects. For example, insufficient lubrication can cause a choppy linear motion or reduce the service life of slide rail guides. Wear and tolerance between assembled parts can result in low positioning accuracy and repeatability. The concept of the compliant mechanism has been introduced into the design of guiding mechanisms to address these common issues due to its merits in high performance and low-cost [3,4].

The compliant mechanism has the capability of transferring or transforming motion, force and energy via the deformation of flexible members [5]. The compliant mechanisms are widely used in the field of surgical robotics [6,7,8,9], positioning stages [10,11,12,13], energy harvesting [14,15,16], mechanical metamaterial [17,18,19,20] and micro-electromechanical system (MEMS) [20,21,22]. Previous researchers have proposed diverse linear guides based on compliant mechanisms. These compliant guides can be classified into planar and spatial linear guides [23].

The parallelogram mechanism is the most common compliant planar guide, and it can perform a single-axis translational movement and has a compact footprint [24]. Generally, the compliant parallelogram mechanisms are composed of two compliant sheets whose ends are fixed to the base and motion stage separately. However, the parallelogram mechanism suffers from parasitic error along the bearing directions.

The parasitic error of the parallelogram can be mitigated by nesting two same mechanisms symmetrically [25]. This mechanism is named as the compound parallelogram mechanism. Furthermore, a fully compliant planar linear guiding mechanism—named XBob—was presented in [26]. The XBob derived from the Roberts four-bar approximate straight line mechanism has the potential use in macro-scale devices or microsystems due to its flat design. In addition, Watt’s mechanism is used as a compliant planar linear guide as well [27].

The diaphragm is one special type of spatial linear guiding mechanism since it is designed flat and can perform an out-of-plane linear movement [28]. Another type of spatial linear guide consisting of folded leaf springs was proposed in [23]. In this design, torsion reinforcement structures are added to increase the stiffness in the bearing directions. Additionally, a cylindrical compliant spring based on the curved parallelogram mechanisms was proposed in [29]. This cylindrical guide benefits from its long stroke, high positioning resolution and movement accuracy. This linear guide is named the detached linear guide in this paper since it comprises the in-parallel curved compound double parallelogram mechanisms (CDPMs) only.

Despite the above benefits existing in the detached linear guide, there are still some open issues, such as significantly reduced bearing-direction stiffness over primary motion. We have improved the detached linear guide by embedding a novel decoupling mechanism between each pair of CDPMs. The design of decoupling mechanisms was inspired by [28].

Applying the decoupling mechanism dramatically improved the bearing-direction stiffness without compromising the stiffness in the motion direction. Furthermore, the issue of bearing-direction stiffness degradation of the detached linear guide is addressed. The decoupling mechanism was developed based on a curved parallelogram mechanism, which deforms along the annular direction.

The methodology used in this paper includes designing a new compliant linear guide mechanism by adding decoupled parallelogram mechanisms [30], nonlinear finite element analysis (FEA) and validation, parametric optimization, prototyping and experimental testing. Nonlinear FEA is used to capture nonlinear characteristics of the design that could not be obtained using the available curved-sheet spatial model (i.e., the linear model as reported in [29,31]) and can also consider the deformation from all structural elements.

This work identifies several main contributions:

- The design of a compliant linear guide (i.e., a decoupled linear guide) that has an improved bearing-direction stiffness without sacrificing its linear stiffness along the motion direction, which is based on connecting decoupling mechanisms to the detached linear guide.
- The comparison of the static stiffness along each axis of decoupled linear guide and that of the detached linear guide via nonlinear FEA simulations.
- A static experimental test conducted on a 3D printed prototype to verify the linear stiffness along the motion direction.

This paper is organized as below. Section 2 proposed the structural design of the cylindrical compliant linear guide with decoupling mechanisms (decoupled linear guide). The deformations of the detached linear guide are shown in this section as well. Section 3 mainly involves the analysis and optimization strategy. A series of nonlinear FEA simulations are performed to discover the nonlinear stiffness before and after applying the decoupling mechanism to the detached linear guide. Then, the decoupled linear guide is optimized and aims to hold a lower primary stiffness.

After determining the geometrical parameters of the detached linear guide, a prototype is manufactured using additive manufacturing technology. An experiment is detailed in this section as well. Lastly, our conclusions are drawn in Section 6.

## 2. Structural Design of Decoupled Linear Guide

The components of the decoupled linear guide proposed in this paper mainly involve the compound parallelogram mechanism, the decoupling mechanism and the centre platform. These three parts form a linear guide together with rigid bodies. The main content of this section is to illustrate the structural design of the decoupled linear guide.

**Figure 1:**
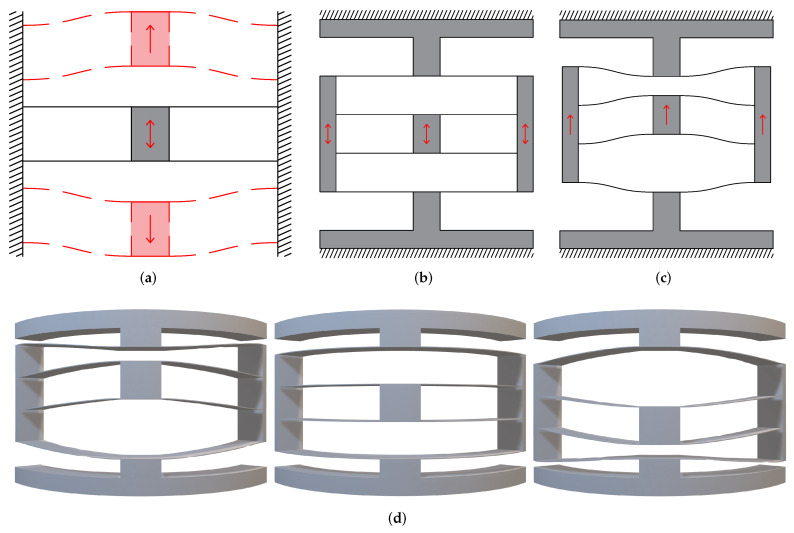
The parallelogram mechanisms. (**a**) The fundamental compound parallelogram mechanism and its deformation. (**b**) Planar CDPM. (**c**) Deformed planar CDPM. (**d**) The deformation process of curved CDPM.

As shown in Figure 1a, the fundamental compound parallelogram consists of a centre shuttle and four straight compliant beams. This mechanism can be regarded as two identical parallelogram mechanisms in a mirror-symmetrical arrangement. The centre shuttle could move in the vertical direction due to the deformation of these beams. Previous guiding mechanisms are primarily designed based on it since they can provide a relatively linear path [32]. However, the stiffness in the desired motion direction is relatively large due to its overconstrained nature.

Hence, the compound double parallelogram mechanism was proposed to solve the problem of large primary stiffness [33]. The structure of the CDPM and its deformed configuration can be seen in Figure 1b,c. The upper and lower surface are fixed. The shuttle of CDPM moves in the vertical direction, which is similar to the motion of the compound parallelogram mechanism. All of the eight compliant beams in CDPM deform, contributing to the movement of the shuttle. The intermediate rigid body also moves vertically during the deformation process. The curved CDPM and its deformation under vertical load is shown in Figure 1d.

Another vital component of the detached linear guide is the decoupling mechanism, which is the part that gives the guiding mechanism higher performance. The decoupling mechanism deforms along the annular direction. The decoupling mechanism is embedded between intermediate rigid bodies of each couple of CDPMs. After applying the decoupling mechanism, the CDPMs in the detached linear guide are connected to form an entire cylindrical structure. When the CDPMs deform, the parasitic motion of the intermediate rigid bodies will tend to cause the decoupling mechanisms to deform.

**Figure 2:**
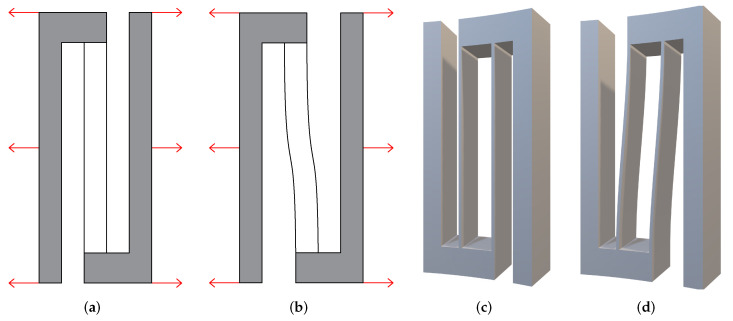
The decoupling mechanism. (**a**) 2D decoupling mechanism. (**b**) Deformed 2D decoupling mechanism. (**c**) 3D decoupling mechanism (load is added in the direction perpendicular to the radius direction). (**d**) Deformed 3D decoupling mechanism.

Figure 2a,b shows the deformation process of the decoupling mechanism in a 2D sketch. The Figure 2c,d illustrate the 3D model of decoupling before and after tension. The tension works along the annular direction, which is perpendicular to the radius direction. The decoupling mechanism keeps its radius constant when it deforms.

**Figure 3:**
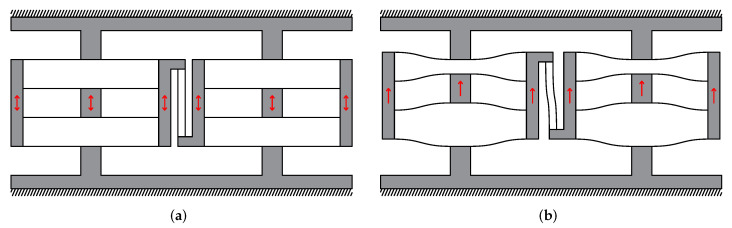
The in-parallel planar CDPMs connected by decoupling mechanism and its deformed shape.

Figure 3a illustrates how to embedding the decoupling mechanism between CDPMs. Figure 3b shows the deformed mechanism where both the CDPMs and decoupling mechanism deform. The compliant beams of CDPM deform in the vertical direction, while the beams of the decoupling mechanism deform transversely.

**Figure 4:**
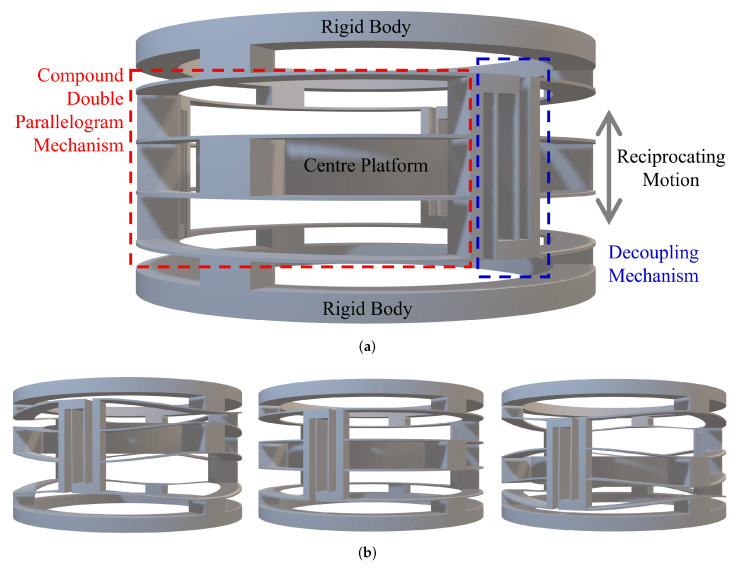
The 3D model of the decoupled linear guide. (**a**) The geometrical design and explanation. (**b**) The deformation process of the decoupled linear guide.

Figure 4a is the front view of the cylindrical compliant guide with three CDPMs. In this case, three in-parallel curved CDPMs are connected by three decoupling mechanisms. A centre platform is fixed to the centre moving shuttle of the CDPM. The moving platform can perform reciprocating motion since this mechanism has only one degree of freedom (DoF). Figure 4b shows the deformation of the entire linear guiding mechanism. This paper assumes the upper and lower annular structures as rigid bodies. The compliant beams are fixed to them.

## 3. Analysis and Optimization

**Figure 5:**
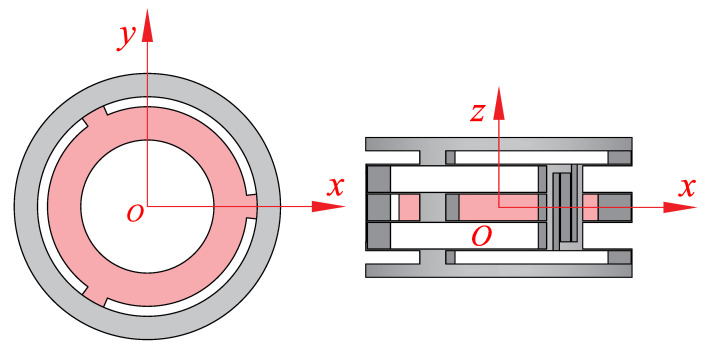
The defined coordinate system.

The main aim of this section is to compare the detached linear guide and decoupled linear guide in the aspect of static stiffness. The performed simulations are based on finite element analysis. The nonlinear simulations reflect the stiffness variation and force–displacement relationships with high accuracy. A global coordinate system is defined to explain the spatial position and arrangement of the model. As seen from Figure 5, the origin of the coordinate system is determined at the geometry centre of the cylinder. The primary motion direction of the decoupled linear guide is defined as the Z-axis. The X-axis and Y-axis are defined based on the right-hand rule.

### 3.1. Nonlinear Stiffness Analysis

The issue of the stiffness degradation of detached linear guide is dealt with by applying the decoupling mechanisms. The stiffness degradation refers to the stiffness in bearing directions that decreases during the deformation process. For the linear guide mechanism, the degradation of stiffness would cause lower precision during the deformation. The centre platform would be easier to swivel or pitch during deformation. Hence, the stiffness degradation should be avoided or weakened.

In this design, we only consider the effects of the geometrical shape on stiffness. The material nonlinearity is not under consideration. The stiffness of the detached linear guide in the bearing directions decreases dramatically when the centre platform moves. The decoupled linear guide addressed this problem by adding the decoupling mechanisms. In addition, the stiffness variation cannot be captured from the linear FEA simulation or linear model. This is because the linear simulation can only produce a linear relationship between the load and displacement, which cannot clearly show the variation process of stiffness. Hence, a series of nonlinear FEA is involved in this part.

The nonlinear simulations further discover the force–displacement relationship and the stiffness-displacement relationship of the compliant linear guiding mechanism. The nonlinear FEA simulation is achieved by the nonlinear static analysis module of Strand7 (R2.4). The geometrical shape of the linear guide is meshed using a rectangular plate element with four endpoints. The maximum length of the mesh edge is fixed as 3% to gain acceptable resolution. The freedom condition of nonlinear FEA is the same as the linear analysis. The upper and bottom rigid bodies are fixed. The constructed points on the centre platform are assigned as one rigid part by using the function called “Auto Assign”.

Then, a mandatory displacement along the motion direction is added to the rigid part. The displacement along the motion direction is set as increasing gradually. The modulus of the material is defined as 3150 MPa, which is obtained from the technical data sheet in [34]. Based on the nonlinear results, the reaction force of the centre platform reflects the magnitude of stiffness. When discovering the stiffness in bearing-directions, the small mandatory translational or rotational displacements are added into the system. The variation of reaction forces in these directions can reflect the stiffness variation.

**Figure 6:**
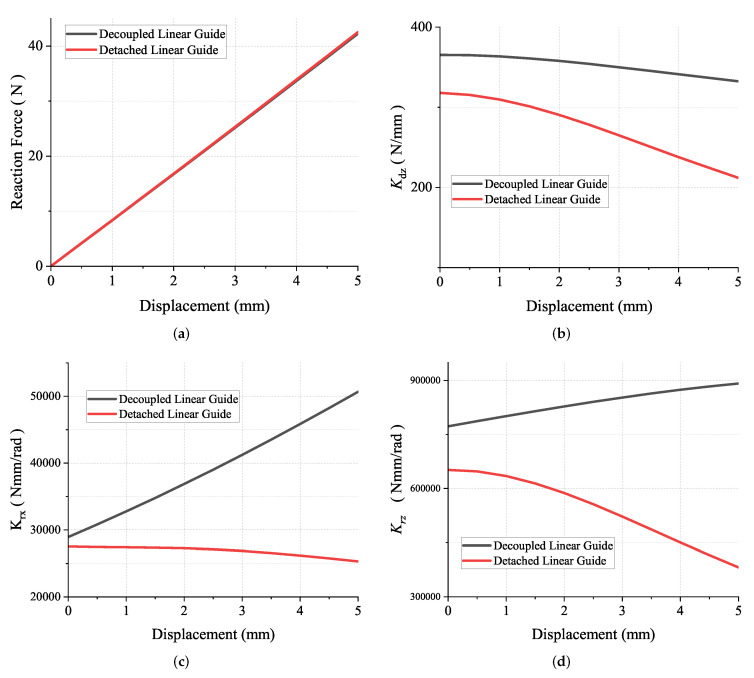
The comparison between the decoupled linear guide and the detached linear guide. The results are derived from the nonlinear FEA simulation. (**a**) The linear relationship between reaction force and displacement along motion direction. (**b**) The translational stiffness along Z-axis. (**c**) The rotational stiffness along X-axis. (**d**) The rotational stiffness along Z-axis.

Figure 6a is the nonlinear simulation result, which shows the relationship between the reaction force in the z-direction and displacement along the motion direction. This figure shows that the difference between decoupled and detached linear guides is tiny. Hence, we can draw a conclusion: adding the decoupling mechanism to the detached linear guide has a small effect on the primary stiffness. Figure 6b shows the relationship between the translational stiffness and displacement along the motion direction.

As can be seen, when the centre platform moves, the stiffness decreases dramatically. This phenomenon is exactly the stiffness degradation, which is mentioned above. After applying the decoupling mechanism to the detached linear guide, the stiffness $K_{dz}$ will not change dramatically. The stiffness degradation of the rotational stiffness along x-axis $K_{rx}$ is also addressed by adding the decoupling mechanism.

The difference is that the $K_{rx}$ of the decoupled linear guide increases instead of decreases. This means that the centre platform of the decoupled linear guide has a higher resistance ability to the rotational external load during the deformation process than does the detached linear guide. In terms of the rotational stiffness about the motion direction $K_{dz}$, the stiffness degradation is addressed as well.

### 3.2. Parametric Optimization

**Figure 7:**
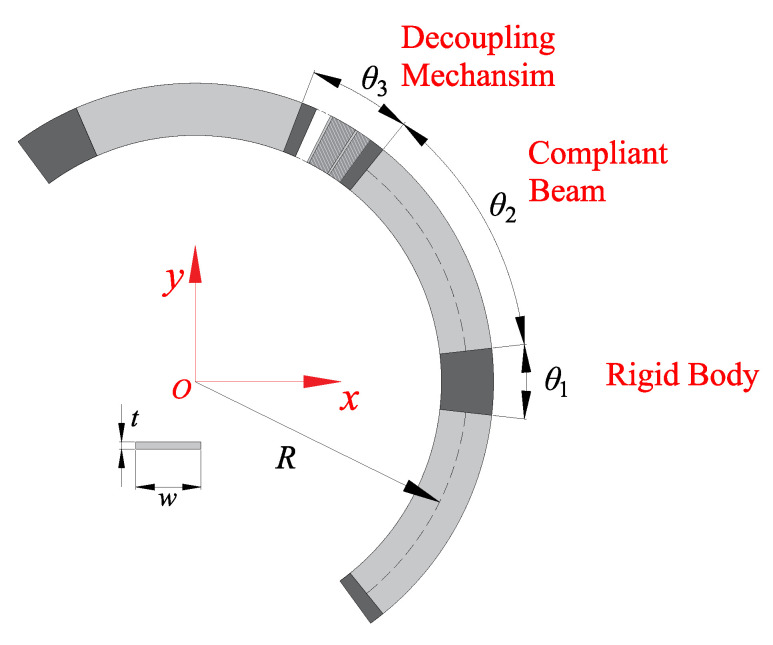
The definition of parameters from the section view of the decoupled linear guide.

In this part, the main aim is to optimize the decoupled linear guide. The optimization aims to find a lower stiffness in the motion direction ($K_{dz}$). The optimization strategy is to change the geometry parameters to obtain a linear guide with lower stiffness. Figure 7 is the section view of the linear guide. The geometry parameters are defined and shown in this figure. The radius *R* represent the average radius of the annulus. The parameter *w* represents the width of the compliant beam. Angles $\theta_{1}$, $\theta_{2}$ and $\theta_{3}$ are defined to illustrate the distribution of rigid block, compliant beam and decoupling mechanism.

The relationship between these angles can be defined as:

(1) $$\theta_{1}+2\theta_{2}+\theta_{3}=\frac{2\pi }{n}$$

where *n* is the number of CDPMs in the detached linear guide. In this design, the number *n* is fixed as 3. Another parameter, “Percentage of the beam $P_{b}$”, is introduced to this system for a more straightforward representation of the proportion of compliant beam. In this design, the $\theta_{1}$ is fixed as $13^{\circ }$ to maintain enough support between the centre platform and compliant linear guide. The Pb can be described as:

(2) $$P_{b}=\frac{\theta_{1}+2\theta_{2}}{\theta_{1}+2\theta_{2}+\theta_{3}}$$

**Figure 8:**
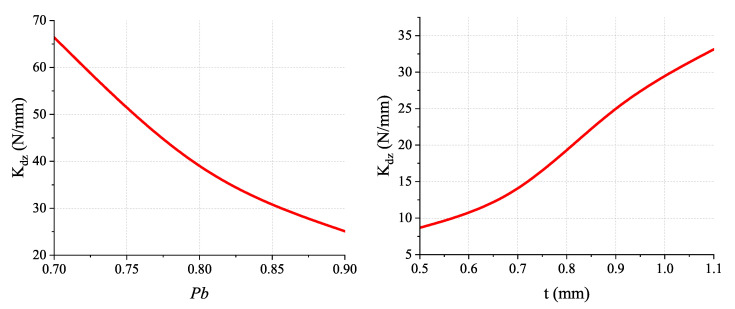

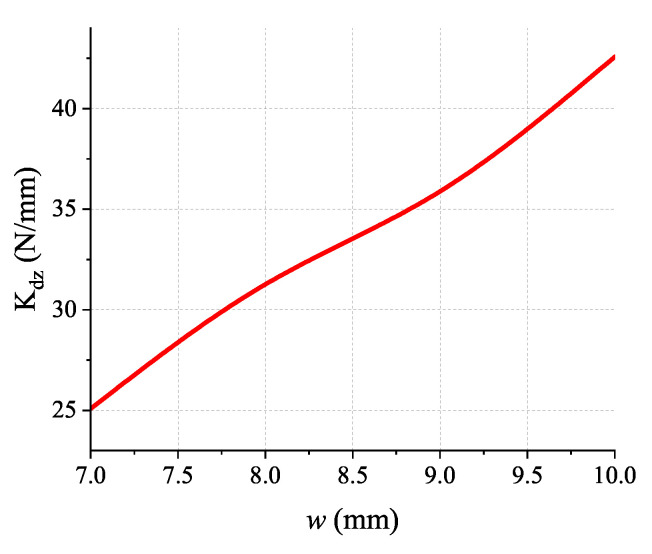
Stiffness under different geometrical parameters.

The geometry radius *R* is fixed as 40 mm in this design. The larger the radius *R* is, the smaller the stiffness $K_{dz}$ is. This compliant mechanism is designed in a compact configuration for trying to suit more application scenarios. Figure 8 shows the main stiffness $K_{dz}$ with different geometrical parameters. The first parameter to be determined is the Pb, which determines the length of the beam and the proportion of the decoupling mechanisms. As can be seen from the results, the higher the Pb is, the lower the stiffness is.

The percentage of the beam is limited to a range from 0.70 to 0.90. This is because, if Pb is too small, the beam would be too short for large range displacement. Furthermore, if Pb is too large, the space for the decoupling mechanism would be insufficient to work properly. Hence, the percentage of beam Pb is determined as 0.90. The next parameter that needs to be determined is the thickness of the beam *t*. Determining the thickness of the beam highly depends on the manufacturing precision. As can be seen from the result, the stiffness $k_{dz}$ increases with the increment of beam thickness. Hence, to obtain a lower stiffness guide, the beam thickness is chosen to be 0.5 mm. Similarly, the width of the compliant beam is chosen to be 7 mm.

## 4. Prototype and Experiment

**Figure 9:**
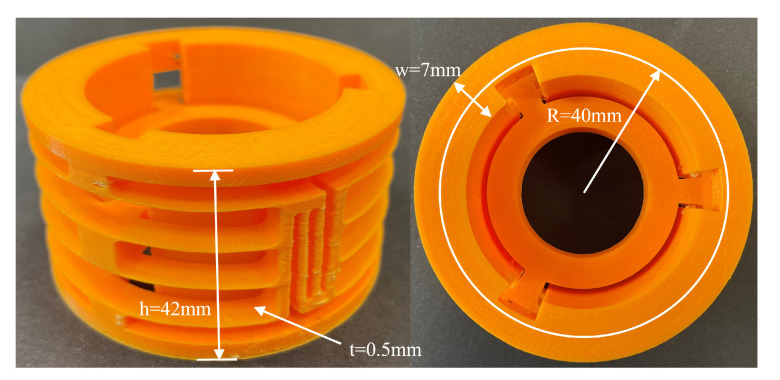
The side view and top view of the printed decoupled compliant guide.

A prototype based on the optimized decoupled linear guide is manufactured using 3D printing technology to verify the FEA analysis. The 3D printer used for prototyping is Ultimaker S3. The slicing software is Ultimaker Cura. The primary material for printing the compliant guide is Polylactide (PLA), and the material used for support structure is Polyvinyl Alcohol (PVA). PVA is one type of water-soluble support material. By applying PVA as the support material, the risk of destroying the thin beams while removing the support structure has been reduced a lot. The print core used for building the primary structure is AA 0.4. The print core used for building the support structure is BB 0.4. The printed model is shown in Figure 9.

The experiment was performed on the texture analyser (TA.Hd plus texture static test system). This system can measure the relationship between load and displacement. The test machine obtains the reaction forces from sensors. In addition, a probe was printed using the 3D printer as well. The probe added a uniform load to the centre platform. To gain an experimental result with higher resolution, the load cell of the texture analyser is selected as 5 kg. The resolution of the force sensor is 0.1 g.

**Figure 10:**
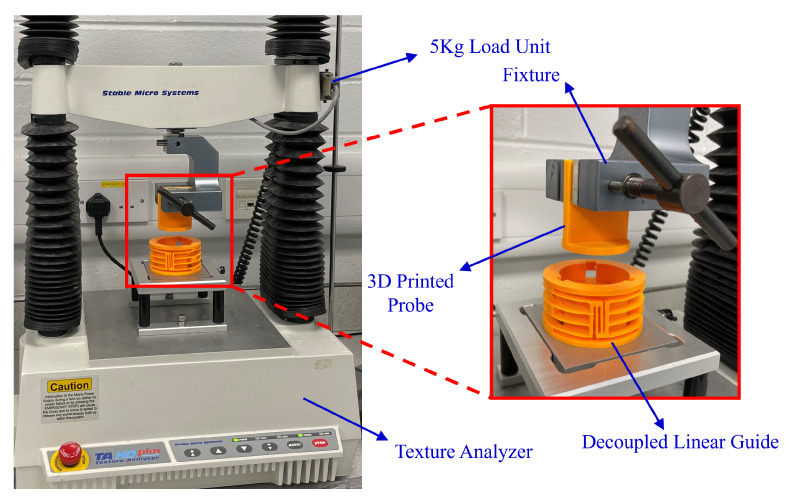
The experimental setup.

In the experiment system, the input is chosen to be displacement along the motion direction. The output is the reaction force. To minimize the dynamic effect, the moving speed is set as 0.1 mm/s. The experimental procedure was repeated five times to gain an average value. The stiffness of the prototype in its motion direction is 1.10773 N/mm. The experiment setup can be seen in Figure 10.

**Figure 11:**
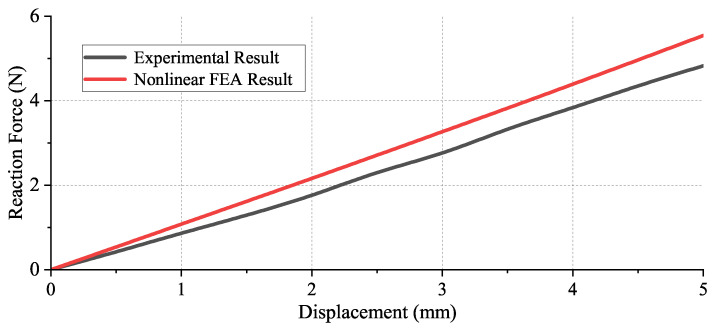
The comparison between the experimental results and nonlinear FEA result.

A comparison between the average experimental results and FEA results is shown in Figure 11. As can be seen, the experimental results are slightly smaller than the FEA results. Several aspects might cause the error. The first aspect is the limited precision of additive manufacturing. The additive manufactured model suffers from orthotropic properties. In addition, the error might be caused by plastic deformation during the repeat deforming process.

## 5. Discussion

The proposed decoupled linear guide offers a new approach for the design of guiding mechanisms.

- First, compared with the conventional slide rail guides [2], the cylindrical shape of the decoupled linear guide makes it more suitable to use than these conventional linear guides in certain scenarios, such as voice coil actuators and electromagnetic energy harvesting. The cylindrical shape ensures the target moving platform moves along its motion direction and avoids wear and friction. The decoupled linear guide has the benefits of lower maintenance costs, high positioning precision and repeatability.
- The decoupled linear guide has a smaller footprint than the spatial diaphragm mechanism [28]. This is because the compliant beams are arranged in a vertical direction instead of a distribution along the radial direction. Furthermore, the decoupled linear guide has higher bearing-direction stiffness than the diaphragm mechanism, which contributes to higher motion precision under payload in bearing directions.
- Compared with the folded leaf springs with torsion reinforcement structures [23], the decoupled linear guide benefits from its simple structure in terms of manufacturing. The application of the decoupling mechanism to improve the bearing-direction stiffness is a different method compared to the use of torsion reinforcement structures for the same motivation.
- Compared to the detached linear guide [29], the primary advantage of the decoupled linear guide is the higher bearing-direction stiffness. They have the same footprint and symmetry.

In summary, the proposed decoupled linear guide has advantages over other linear guides, such as assembly issues, positioning precision and manufacturing complexity. A summarized table is shown in Table 1, which illustrates the comparison between different compliant spatial linear guides.

## 6. Conclusions

In this work, we presented a novel cylindrical compliant linear guide with decoupling mechanisms. The compliant-mechanism-based linear guide has the benefits of no friction, backlash, wear and high precision. In addition, a decoupling mechanism was designed and embedded in the detached linear guide. The nonlinear FEA showed that the decoupled linear guide had higher bearing-direction stiffness while keeping the same primary stiffness. Furthermore, the nonlinear FEA simulations proved that the new design addressed the problem of stiffness degradation as can be seen in most reported compliant linear guide. Finally, the static experimental test was performed on a 3D printed prototype and showed that the stiffness in the motion direction was linear.

The main outcomes of this paper are listed below:

- The decoupled linear guide had higher stiffness compared with the detached linear guide in all the bearing directions during deformation.
- As proved from nonlinear FEA, the stiffness degradation of the detached linear guide was addressed by adding the decoupling mechanisms.
- A prototype of the decoupled linear guide was manufactured where the stiffness in its motion direction was 1.0073 N/mm. The stiffness derived from the nonlinear FEA result was 1.1162 N/mm. The error between the FEA and experimental results was 9.76%.

The main limitation of this paper is the absence of a nonlinear mathematical model. The mechanism can be better explained by modelling the relationship between force and displacement, which forms part of our future work. Applications of the decoupled linear guide will also be investigated in some promising fields, such as voice coil actuators and electromagnetic energy harvesting.

## Figures and Tables

**Table 1:** The summarized comparison between types of compliant spatial linear guides.

	Linear Stiffness in Motion Direction	Alleviated Bearing-Direction Stiffness Reduction	Symmetrical Design for Eliminating Parasitic Motion	Compactness	Simple Manufacturing
Decoupled Linear Guide	✓	✓	✓	✓	✓
Spatial Diaphragm [28]	✓		✓		
Folded leaf springs [23]	✓	✓	✓		
Detached Linear Guide [29]	✓		✓	✓	✓
